# University Students’ Successive Development From Entrepreneurial Intention to Behavior: The Mediating Role of Commitment and Moderating Role of Family Support

**DOI:** 10.3389/fpsyg.2022.859210

**Published:** 2022-03-29

**Authors:** Hu Mei, Zicheng Ma, Zehui Zhan, Wantong Ning, Huiqi Zuo, Jinbin Wang, Yingying Huang

**Affiliations:** ^1^Key Lab for Behavioral Economic Science and Technology, School of Economics and Management, South China Normal University, Guangzhou, China; ^2^Management College, Guangdong Polytechnic Normal University, Guangzhou, China; ^3^School of Information Technology in Education, South China Normal University, Guangzhou, China; ^4^Key Laboratory of Brain, Cognition and Education Sciences, South China Normal University, Ministry of Education, Guangzhou, China

**Keywords:** successive development, entrepreneurial intention, entrepreneurial commitment, family support, entrepreneurial behavior

## Abstract

University students having high entrepreneurial intention while not transferring into actual entrepreneurial behavior is a contradictory issue in need of in-depth research. To explore the successive development mechanism of the entrepreneurial process, this study constructed a moderated mediation model to examine whether entrepreneurial commitment from three dimensions (affective, behavioral, and continuance) mediated the relationship between entrepreneurial intention and behavior, and whether this mediating process was moderated by family support. A survey was conducted among university students from six major universities in south China using the snowball sampling approach. A total of 469 valid responses were obtained (44.6% male and 55.4% female participants). Structural equation modeling was adopted for data analysis. According to the results of the confirmatory factor analysis, it was found that entrepreneurial intention had both direct and indirect positive effects on entrepreneurial behavior, while entrepreneurial commitment worked as the mediator, and family support moderated the relationship between entrepreneurial intention and behavior. Results indicated that entrepreneurial commitment bridged the path from entrepreneurial intention to behavior, and family support created the boundary effect. This finding highlights the importance of guiding students through entrepreneurial commitment toward entrepreneurial behavior, and pays special attention to the crucial role of family support under the national strategy.

## Introduction

Entrepreneurship plays a key strategic role in global economy (Chandra, 2018; Dhahri and Omri, 2018), job creation (Kim et al., 2018), business opportunities (Global Entrepreneurship Monitor [GEM], 2022), social impact, and particularly during global crisis such as the COVID-19 pandemic (Sieger et al., 2021). In recent years, the GUESSS project (i.e., global university enterprise spirit students’ survey) has attracted wide attention, and reflected great importance on university students’ entrepreneurship research. Many countries and governments have successively enacted a series of policies on finance, taxation, and education (Liao et al., 2017) for fostering entrepreneurship among university students. However, there is still a big gap between students’ entrepreneurial intention and their entrepreneurial behavior. According to relevant Chinese survey results, up to 70–80% of university students have reported entrepreneurial intention, while only 0.3–2% of them have actually engaged in entrepreneurship (Zhu et al., 2017). The Sieger et al. (2021) showed that the current transformation of global university students’ entrepreneurial intention into behavior was not optimistic, with 50.1% of all students (*N* = 75’838) intended to be an entrepreneur, but only 28.4% of all students actually started up their business. The individual behavior in entrepreneurship is usually the purpose and destination of research. However, due to the complexity and difficulty of measuring behavior, after Bird (1988) creatively proposed that entrepreneurial intention was a prerequisite for the entrepreneurial behaviors, researchers began to study entrepreneurial intention as the starting point of entrepreneurial process to represent behaviors (Bird, 1988; Douglas and Fitzsimmons, 2013). In essence, entrepreneurial intention is the entrepreneurial idea aiming at planned behaviors (Ajzen, 1991; Krueger et al., 2000). Only the talents with entrepreneurial intention can start their entrepreneurial behaviors (Thompsone, 2009). China and some emerging countries have conducted a large number of studies on entrepreneurial intention and its transformation to entrepreneurial behavior (Cui et al., 2017; Bogatyreva et al., 2019). However, later on, scholars started to realize the drawbacks of using entrepreneurial intention to predict behaviors. A meta-analysis showed that intention can only explain 28% of the variation of behavior (Sheeran, 2002), so the “jump” prediction of behavior by entrepreneurial intention is not reasonable (Shirokova et al., 2016; Ma, 2017). Shirokova et al. (2016) suggested that although many studies confirmed the high correlation between intention and behavior, however, the transformation from intention to behaviors is indirect and with uncertainty. The uncertainty and complexity of the entrepreneurship process has caused the deviation of entrepreneurial behavior from intention and a cognitive bias in entrepreneurship cognition. These deviations leads to the ultimate failures in transforming entrepreneurial intention into real behavior (Adam and Fayolle, 2015). Bird et al. (2012) made a pertinent comment on entrepreneurial behavior research and claimed that entrepreneurs’ behavior is affected by cognition and emotion. What we see is only the appearance of behavior. In fact, the invisible cognitive causes of behavior are prone to be more interesting. In order to solve this “gap,” Gollwitzer (1999) suggested that entrepreneurial commitment is a psychological variable that is more observable than entrepreneurial intention, but has not yet been performed as behavior. Entrepreneurial commitment plays an intermediary role in the relationship between entrepreneurial intention and entrepreneurial behavior (Fayolle et al., 2011). If the entrepreneurs are willing to invest a high degree of time, energy, money, intelligence, and endurance in entrepreneurship, rather than just intention, they are more likely to implement entrepreneurial behavior (Fayolle and Liñán, 2014; Esfandiar et al., 2019). Accordingly, we propose a basic entrepreneurial cognition model of “intention–commitment–behavior.” The above views provide a new perspective for linking between entrepreneurial intention and entrepreneurial behavior.

The present study constructed a model from entrepreneurial intention to behavior to fill the gap, and tried to answer the following research question: How does university students’ entrepreneurial attention affect their entrepreneurial behaviors in the Chinese context? In terms of the internal factors, entrepreneurial commitment is proposed as a psychological variable closer to entrepreneurial behavior (Fayolle et al., 2011; Vamvaka et al., 2020). This study explores the transformation from intention to behavior through commitment by testing its mediating effect.

Besides, our aim was to determine the most important external factors influencing students when making decisions in the Chinese context. Thus, we conducted an investigation among university students on “*The person who has most influenced you in your decision-making process*.” Among the 124 questionnaires, 88 respondents (71%) reported that parents or siblings had the greatest impact on their major decisions. Results indicated that family support is the most important factor, which we analyzed in this study considering its crucial role in decision-making for inexperienced Chinese university students. In fact, lots of research confirmed that family background affected students’ entrepreneurial intention (Herman, 2019; Huang, 2021). Family support plays a positive regulatory role in Chinese farmers; entrepreneurship (Dong and Zhao, 2019; Yang et al., 2019) and university students’ entrepreneurship (Tian and Chen, 2019). Its moderating effect has been tested to investigate the boundary conditions of intention, commitment, and behavior. This study aspired to make two main contributions. First, an entrepreneurial cognitive model, based on “intention–commitment–behavior,” is proposed in this paper. It provides a new perspective for understanding the entrepreneurial psychological process of university students and deepens the research on “entrepreneurial commitment” to a certain extent. Due to the important role of “family support” in decision-making, the moderating effect of “family support” on “entrepreneurial intention to behavior” partly reflects the characteristics of university students’ entrepreneurship in the Chinese context. Second, this study focuses on the bridging role of “entrepreneurial commitment” and highlights the important role of family support in entrepreneurship in China. It also provides guidance for cultivating entrepreneurial talents and improving the diversification of entrepreneurship education in practice.

## Theory and Hypotheses

### Entrepreneurial Intention and Entrepreneurial Behavior

Entrepreneurial intention is a psychological state of entrepreneurs when they start a new venture or create new values in an extant enterprise (Bird, 1988). It is effectively predictable for such a rare, unobservable and time-lagged activity as entrepreneurship (Krueger et al., 2000). Entrepreneurial behavior involves specific activities of individuals inspired by the idea of starting a business (Penrose, 1959), and it has both a narrow and a broad sense. Narrow entrepreneurial behavior emphasizes the entrepreneurial opportunity identification and resources integration throughout the entrepreneurial process, while the broad sense includes a series of behaviors from survival to development after starting a business. We explore the entrepreneurial behavior from a narrow sense in the following sections.

Despite entrepreneurial behavior being the final goal of entrepreneurial intention (Gieure et al., 2020), due to the inherent difficulty of entrepreneurial behavior research, scholars did not focus on the antecedent variables of entrepreneurial behavior until Bird (1988) creatively proposed entrepreneurial intention as the precondition for starting and developing a new business in the late 1980s. Most of them noticed entrepreneurial intention as the starting point of the entrepreneurial process (Newman et al., 2019). Thereafter, the application of Planned Behavior Theory in entrepreneurship research has further stated the role of entrepreneurs’ attitudes (Barrios et al., 2021), namely, entrepreneurial intention, on their actual behavior (Ajzen, 1991; Mei et al., 2016). Essentially, entrepreneurship is intentional in view of entrepreneurial intentions as the entrepreneurial ideas aiming at planned behavior (Krueger et al., 2000). Thus, entrepreneurial intention is the only best predictor of entrepreneurial behavior (Krueger, 2017).

Individuals with high entrepreneurial intention are more concerned and sensitive to entrepreneurial information (Farrukh et al., 2017). They always have a stronger desire to achieve their entrepreneurial goals than those with low intentions (Bird, 1988). Specifically, they are inclined to respond sensitively to the related information (Kickul et al., 2010), in order to identify entrepreneurial opportunities quickly (Fearon et al., 2019) and skillfully integrate external entrepreneurial factors, such as materials, technology, and information (Meyer and Meyer, 2020). Meanwhile, their desire for entrepreneurial goals will be internalized as the motivation to inspire themselves to work hard toward achieving the presupposed goals (Sheeran, 2002). Only intrinsic motivation can have a positive influence on individuals (Ryan and Deci, 2020; Zhan et al., 2021a). If the antecedents of entrepreneurship are met, they would implement entrepreneurial behavior and advance on the right track (Barrios et al., 2021). Accordingly, this paper suggests that entrepreneurial intention has a positive effect on the development of entrepreneurial behavior. Thus, based on the arguments presented above we hypothesize:

H1: Entrepreneurial intention will be positively related to entrepreneurial behavior.

### Entrepreneurial Intention, Commitment, and Behavior

Multiple studies have argued that entrepreneurial intention does not transform directly into entrepreneurial behavior (Sheeran, 2002; Zhu et al., 2017), and the entrepreneurial cognitive bias has aroused some scholars to seek the bridge between them. Hereby, drawing upon the concept of organizational commitment with three components affective, normative and continuance (Meyer and Herscovitch, 2001), entrepreneurial commitment has been proposed and considered a closer psychological variable to entrepreneurial behavior. If individuals intend to be self-employed and are willing to devote a huge amount of time (Wood et al., 2019), energy (Naz et al., 2020), money (Bice, 2020), intelligence (Ristianti et al., 2020), and endurance (Barba and Atienza, 2017) to entrepreneurial activities, they would have high entrepreneurial commitment and are more likely to start up rather than only staying with intentions. Therefore, entrepreneurial commitment plays a role as bridge between “intention” and “behavior.” In addition, entrepreneurial commitment could be divided into three key dimensions: affective, behavioral, and continuance commitment (Tang, 2008).

Actually, individuals with higher intentions would be more sensitive and concerned about information related to entrepreneurship, such as that from entrepreneurial books, contests, study classes (Hassan et al., 2020), experience sharing meetings (Giones et al., 2016), business incubators (Al-edenat and Hawamdeh, 2020), policies (Bahl et al., 2020), and so forth. With unconscious influence, they will be determined to pursue their entrepreneurial intention with more motivation and willingness to promote entrepreneurial ideas, and naturally form the desire to start businesses, which is also known as entrepreneurial commitment. It is an individual’s internal commitment to undertake entrepreneurial activities in the future. Those with high entrepreneurial commitments spend more time studying entrepreneurial knowledge (Saptono et al., 2020), which means they are more likely to choose to start businesses in the future. Though they might fail, they would keep going until they succeed. There is no doubt that entrepreneurial commitment binds an individual to their goals (Fayolle and Liñán, 2014; Dahmardeh and Nastiezaie, 2019). Therefore, it is considered as a bridge between entrepreneurial intention and behavior (Wallmeroth et al., 2018), playing its transitional role between them. The above explains the “intermediary role” of “entrepreneurial commitment” to a certain extent. In conclusion, we thus propose:

H2: Entrepreneurial intention will be positively related to entrepreneurial commitment.

H3: Entrepreneurial commitment will be positively related to entrepreneurial behavior.

H4: Entrepreneurial commitment will mediate the relationship between entrepreneurial intention and behavior.

To understand the specific role of entrepreneurial commitment, we test the mediating effect of entrepreneurial commitment from its three dimensions. We adopt a pattern most accepted by scholars to divide its dimensions into affective commitment, behavioral commitment, and continuance commitment (Tang, 2008; Indrawati et al., 2015). Specifically, affective commitment notes the willingness, excitement, and persistence of individuals in entrepreneurship (Jena, 2020), which also shows individuals’ psychological attachment to the employment through fondness, pleasure, or preference (Zhang et al., 2019). As for those who regard entrepreneurship as the enactment of a non-pecuniary goal (Dahmardeh and Nastiezaie, 2019), a high level of commitment will facilitate the continuance of their venture to realize their own goals. Meanwhile, people with high levels of behavior commitment focus more on accomplishment of their entrepreneurial “task”; hence, they are willing to devote whatever they have to the activity (Tang, 2008), such as time, money, effort, and passion. Continuance commitment is closely related to the costs of giving up their present position (Meyer and Allen, 1991). Based on some studies of commitment (Tremblay, 2021), it is twofold: first, people with a high level of continuance commitment resist stopping halfway considering the huge costs and sacrifices; second, they will not recognize other options as an alternative in their career. This study adopted the three-component model of commitment accepted by Tang (2008) and Indrawati et al. (2015) to test the mediating effect of three underlying dimensions of entrepreneurial commitment. Hence, hypothesis H4 also includes the three following sub-hypotheses:

H4a: Affection commitment will have a mediating effect on the relationship between entrepreneurial intention and behavior.

H4b: Behavior commitment will have a mediating effect on the relationship between entrepreneurial intention and behavior.

H4c: Continuance commitment will have a mediating effect on the relationship between entrepreneurial intention and behavior.

### The Moderating Effect of Family Support

Family plays an essential role in shaping an individual’s propensity for aspects of emotion (Edelman et al., 2016), spirit (Abraham, 2020), and mentality (Saptono et al., 2020). It is the primary and most powerful emotional system for an individual (Xu et al., 2020), and parents provide the most support for the young in making occupational decisions. We argue that the decisive role of parents dominates personal decisions in the Chinese context according to our survey of 124 university students, of whom 81 (65.3%) responded parents and 7 (5.6%) chose brothers or sisters in answer to the question: “The person who has most influenced you in your decision-making process.” Due to the complexity of self-employment, which is one of the most important career choices for contemporary university students, their families have certain expectations and make requests. Family support of emotions or resources can help students to enhance their abilities and confidence in dealing with difficulties, construct their mental safeguards (Zhong et al., 2016), meet uncertainty and emergency issues (Zhan et al., 2021b,c), and make strategic decisions in a calmed state under low pressure (Zhang et al., 2019). Specifically, family members would take an approach to offer entrepreneur resources with lower prices, because of their close relationship and inherent consciousnesses (Banerji and Reimer, 2018). Besides, young entrepreneurs who are resource-poor always ask those whom they have strong ties with for help throughout the emergence phase (Klyver et al., 2018; Abraham, 2020). Not only that, when entrepreneurs want to share some ideas, their parents are the best listeners. Similarly, the suggestions of parents are vital for those entrepreneurs who need to make an important decision (Annisa et al., 2021). Consequently, throughout the entrepreneurial process, family support mainly indicates the support that parents offer to entrepreneurs, such as entrepreneurial funds, information, connections, and emotion (Edelman et al., 2016; Jena, 2020).

This study suggests that entrepreneurs with high family support can receive more understanding and respect from parents for their engagement in entrepreneurship and the special occupational decision they have made undertaking more risk and responsibility (Verver and Koning, 2017). The parents might support them in terms of venture capital, interpersonal networks (Bohlmann et al., 2017), work experience (Estrada-Robles et al., 2020), and care to ease their stress when making strategic decisions in a complicated and changeable social environment (Zhang et al., 2019). That is, family support would help to transform entrepreneurial intention into behavior (Jaskiewicz et al., 2015; Hernández-Linares and López-Fernández, 2018). In addition, the increase in their entrepreneurial commitment after overcoming the main obstacles in entrepreneurship also speed up the translation of entrepreneurial behavior (Failla et al., 2017). Furthermore, those with high entrepreneurial intention and family supports might have a strong sense of moral obligation and proceed to entrepreneurial plans and behaviors in return for family support (Edelman et al., 2016). On the contrary, parents of those with low family supports lack understanding of their work and even hinder them, so that their sense of frustration would increase tremendously while their possibility of success would decrease. Based on this, the following hypotheses were proposed:

H5: Family support will have a positive moderating effect on the relationship between entrepreneurial intention and behavior.

H6: Family support will have a positive moderating effect on the relationship between entrepreneurial intention and commitment.

H7: Family support will have a positive moderating effect on the relationship between entrepreneurial commitment and behavior.

**FIGURE 1:**
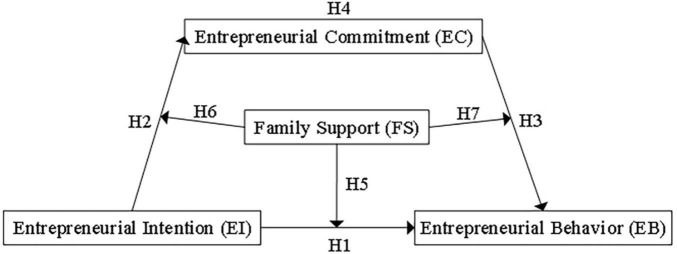
Conceptual model.

The conceptual model of the action mechanism of entrepreneurial intention to engage in entrepreneurial behavior in this study is shown in Figure 1.

## Materials and Methods

### Participants

In this study, snowball sampling approach was utilized to recruit university students from six major universities in South China and encourage them to pass the survey on to other students. The snowball sampling approach is often used for the survey of rare groups, such as university students. First find an individual (i.e., the “source,” also referred to as the “seed”) who has the desired characteristics and uses the person’s social networks to recruit similar participants (Sadler et al., 2010). A total of 521 questionnaires were distributed and 495 respondents were obtained, including 469 valid respondents, the recall rate was 95%. Missing values were specified for the questionnaires with fewer missing values, and holographic maximum likelihood estimation was used. The overall effective rate was 94.75%. The respondents of the questionnaire survey consisted of 55.4% females; 7.5% were aged 18 years old or below, 90.8% aged between 19 and 22 years old, and 1.7% aged 23 years old or above; 20.9% were freshmen, 27.3% were sophomores, 29.4% were juniors, and 22.4% were seniors. Regarding the occupation of the respondents’ fathers, privately- or individually-owned business accounted for 28.6%, followed by farmers (16.0%) and workers (14.9%). Regarding the occupation of the respondents’ mothers, most mothers were from other occupations, accounting for 23.8%, followed by privately- or individually-owned business (21.4%) and farming (16.2%). Additionally, the highest ratio (23.1%) of family per capital monthly income was located in the range of 3,500–6,000 RMB.

### Measures

The chosen constructs are mostly based on established measurement scales. In line with relevant research (Edelman et al., 2016; Liu T. et al., 2019), we conceptualized and measured the family support with 12 items, such as “*My parents respect my idea of starting a business*.” Apart from the measurement of family support compiled by the research team, other variables were mainly based on the mature scale in China and other countries. Gordon approach was adopted to determine the number of entries and the representation of the Family Support Scale. The contents are as follows:

*Entrepreneurial intention* was operationalized as construct with four formative dimensions (Mei et al., 2016, 2017; Hoang et al., 2020), capturing different configurations that might promote the innovation behavior, that is, uncertain timetable, unlimited timetable, limited timetable, and clear timetable.

*Entrepreneurial commitment* was assessed with a measurement scale based on Iffan (2018), with 10 items, comprising affection commitment (items 1–4), behavior commitment (items 5–9), and continuance commitment (items 9–10).

*Entrepreneurial behavior* was assessed with 15 items extracted from Edelman et al. (2016) and Vamvaka et al. (2020), referring to behavior about ‘knowledge preparation,’ “ability to cultivate,” “team preparation,” “information preparation,” “fund preparation,” and “relationship preparation.”

*Family support* was assessed with a validated scale with 12 items developed by Edelman et al. (2016) and Liu T. et al. (2019), which reflected the degree of parental support for the respondents’ entrepreneurship.

We controlled family location, parent’s occupation, family income, and entrepreneurial experience of family member and friends as potential control variables.

Unless otherwise stated, the participants indicated their agreement on a 5-point Likert scale (from 1 = strongly disagree to 5 = strongly agree). The final list of items for each construct is shown in Table 1. The specific contents of constructs and their reliability and validity are shown in Table 2.

**TABLE 1:** Summary of fit indices.

Variable	χ*^2^*	*df*	χ*^2^*/*df*	CFI	TFI	RMSEA
Entrepreneurial intention	6.734	2	3.367	0.988	0.965	0.071
Entrepreneurial commitment	85.691	32	2.678	0.973	0.963	0.060
Entrepreneurial behavior	103.669	27	3.840	0.955	0.940	0.078
Family support	102.211	27	3.786	0.950	0.933	0.077

**TABLE 2:** Reliability and validity.

Variable	Measure indexes	Loading
Entrepreneurial intention	EI1 I think I will start a business in the future	0.618
CR = 0.752	EI2 If I have the chance to make the decision by myself, I will choose my own business	0.612
	EI3 Considering all sorts of restrictions (such as lack of funds, family opposition, etc.), I would still prefer to start business	0.675
	EI4 It is likely that I will start my own business in the next 5 years	0.718
Entrepreneurial commitment	EC1 I prefer to start my own business compared to other career options	0.799
CR = 0.930	EC2 Starting a business will help me achieve other important goals in my life	0.775
	EC3 I will put my heart and soul into the process of entrepreneurship	0.871
	EC4 I think entrepreneurship is promising I am willing to go on	0.834
	EC5 I will try my best to starting my business	0.759
	EC6 I would like to choose to start business, even if I have to do many things like customer visits or propaganda every day	0.819
	EC7 I’d be happy to work more than fifteen hours a day for my goals	0.660
	EC8 I will actively build up my own social relationship and try to get support from all sides	0.655
	EC9 If this business idea turns out to be fail, I will choose to get employment	0.687
	EC10 As the business has put a lot of effort into it, I won’t consider abandon	0.660
Entrepreneurial behavior	EB1 I’ve had a lot about entrepreneurial courses	0.614
CR = 0.887	EB2 I often attend lectures on business administration or entrepreneurship	0.628
	EB3 I’ve been participated in many entrepreneurship competition	0.643
	EB4 I have establish bantam store and enterprise	0.619
	EB5 I’ve got some appropriate business partners	0.716
	EB6 To gain in-depth information about the market, I’ve done a lot of research	0.739
	EB7 I have taken my initiative to understand the process of entrepreneurship	0.725
	EB8 I have successfully raised the funds for starting a business	0.735
	EB9 I have set up a social network for business need	0.722
Family support	FS1 My parents respect my idea of starting a business	0.699
CR = 0.873	FS2 My parents believe that I can succeed in entrepreneurship	0.683
	FS3 My parents are concerned about my preparation for Entrepreneurship	0.661
	FS4 My parents are willing to provide me with start-up funds	0.687
	FS5 My parents will advise me on entrepreneurship	0.672
	FS6 My parents will help me make my entrepreneurial decision	0.631
	FS7 My parents will look for solutions to the problems I have encountered in the process of entrepreneurship	0.633
	FS8 My parents will use their personal connections to help me find opportunities for Entrepreneurship	0.611
	FS9 My parents think that entrepreneurship can test my ability	0.647

### Data Collection and Analysis

SPSS 20.0 was used to run exploratory factor analysis (EFA) with half of the collected data. The KMO value was 0.821, and the Bartlett test was significant (*p* < 0.001). Six items for entrepreneurial behavior and three for family support were removed, as the results implied a low-level reliability load (less than the threshold of 0.5).

In the other half of the sample, the structural equation modeling was used for confirmatory factor analysis. The results showed that the model was well fitted with the data, and the specific results are demonstrated in Table 1, indicating that the factor load of each measurement index was higher over 0.5. Table 2 illustrates that the range of Cronbach’s alpha of each variable is 0.752–0.884, reaching more than the threshold of 0.7, and the combination reliability (CR) of each factor was greater than 0.7, indicating that the measurement has good reliability.

The descriptive statistical results of the variables are shown in Table 3, and the correlation analysis shows that there is a significant positive correlation between entrepreneurial behavior and entrepreneurial intention, entrepreneurial commitment, and family support (*p* < 0.001). It can be seen that the correlation between the variables is basically consistent. The variance expansion factor (VIF) of each variable is far less than 10, which indicates that there is no serious multi-collinearity between variables. The square roots of each factor average variance extracted are greater than the other related line values of its row and column, and the discriminant validity of the scale is good.

**TABLE 3:** Correlation analysis.

Variable	Mean	*S.D.*	*V1*	*V2*	*V3*	*V4*	*V5*	*V6*	*V7*	*V8*	*V9*	*V10*	*V11*	*V12*
Entrepreneurial interest	3.19	0.812												
Family location	3.07	1.385	0.091											
Father’s occupation	4.82	1.973	0.024	0.216**			.							
Mother’s occupation	5.35	2.047	–0.016	0.087	0.514**									
Father’s education	2.09	1.529	–0.082	−0.259**	−0.278**	−0.178**								
Mother’s education	1.88	1.520	−0.117*	−0.212**	−0.225**	0.024	0.697**							
Family monthly income per capita	2.91	1.568	–0.015	−0.336**	−0.189**	–0.016	0.187**	0.210**						
Family’s entrepreneurial experience	1.53	0.499	−0.128*	0.140**	–0.025	–0.082	0.037	0.086	−0.225**					
Friends’ entrepreneurial experience	1.39	0.488	−0.134*	–0.002	–0.032	−0.117*	0.046	0.031	–0.016	0.274**				
Entrepreneurial intention	2.56	0.774	0.394**	0.077	−0.106*	–0.015	–0.071	0.012	–0.026	−0.120*	−0.143**			
Entrepreneurial commitment	2.92	0.682	0.306**	0.076	−0.095*	−0.128**	–0.086	–0.010	0.022	–0.042	–0.040	0.453**		
Family support	2.09	0.794	0.100*	–0.005	−0.147**	−0.134**	0.025	0.067	0.108*	–0.032	–0.061	0.416**	0.456**	
Entrepreneurial behavior	2.79	0.812	0.193**	–0.092	–0.034	0.394**	0.076	0.041	0.117*	−0.143**	−0.122*	0.315**	0.174**	0.286**

*p < 0.05, V1, entrepreneurial interest; V2, family location; V3, father’s occupation; V4, mother’s occupation; V5, father’s education; V6, mother’s education; V7, family monthly income per capita; V8, family’s entrepreneurial experience; V9, friend’s entrepreneurial experience; V10, entrepreneurial intention; V11, entrepreneurial commitment; V12, family support; V13, entrepreneurial behavior.

## Results

### Common Method Bias

This study performed the Harman’s one-factor test (Podsakoff and Organ, 1986) to examine the common method bias. The results reveals that, no single factor emerged from this analysis, nor was there a general factor that was greater than 40% of variance in these variables. The first factor explained only 26.055% of the total variance. Thus, this indicates that common method bias is not an issue in this study.

### Testing of the Mediating Effect

We performed structural equation modeling using Mplus 7.4 to test the hypotheses. Firstly, we tested the effect of the predictor variable on the outcome variable. Results showed that entrepreneurial intention had a significantly positive effect on entrepreneurial behavior (γ = 0.519, *p* < 0.001). The mediating model (M_e_) fit indices (χ^2^/*df* = 2.169, χ^2^ = 676.834, *df* = 312, RMSEA = 0.052; SRMR = 0.071; CFI = 0.918, TLI = 0.909) all met the requirements of the study, indicating good fit to the sample data. Then, we tested the significance of the two mediating path coefficients. Results indicated that entrepreneurial intention had a positive effect on entrepreneurial commitment (α_me_ = 0.625, *p* < 0.001). Entrepreneurial commitment also had a significant, positive relationship with entrepreneurial behavior (β_me_ = 0.450, *p* < 0.001). Therefore, it was concluded that the mediating effect of entrepreneurial commitment reached a significant level. Lastly, after introducing entrepreneurial commitment as a mediating variable, the direct effect of entrepreneurial intention as a predictor on entrepreneurial behavior as an outcome variable was still significant (γ_me_ = 0.256, *p* < 0.001). Thus, the results provide support for H1, H2, and H3, demonstrating that entrepreneurial commitment plays a partial mediating role between entrepreneurial intention and entrepreneurial behavior, which supports H4.

Further, we examined the effects of three sub dimensions of the mediator variable using the same tests as above. The mediating models of affection (M_e1_), behavior (M_e2_), and continuance (M_e3_) revealed the following good fit to the data: χ*^2^*/*df* = 2.621, 2.140, 2.249 (χ*^2^* = 471.851, 385.178, 321.666, *df* = 180, 180, 143); RMSEA = 0.061, 0.051, 0.053; SRMR = 0.076, 0.067, 0.062; CFI = 0.916, 0.929, 0.925; TLI = 0.905, 0.920, 0.913. It was found that entrepreneurial intention had a positive significant impact on affection, behavioral, and continuance commitment, respectively (α_e1_ = 0.530, *p* < 0.001; α_e2_ = 0.333, *p* < 0.001; α_e3_ = 0.424, *p* < 0.001), and these three dimensions of commitment also positively influenced entrepreneurial behavior (β_e1_ = 0.197, *p* < 0.01; β_e2_ = 0.314, *p* < 0.001; β_e3_ = 0.161, *p* < 0.05). Therefore, we determined that the mediating effects of affective, continuance, and behavioral commitment reached a significant level. Lastly, after introducing these three mediator variables in the model, the result still revealed the significant effect of entrepreneurial intention as a predictor on the outcome variable entrepreneurial behavior (γ_e1_ = 0.427, *p* < 0.001; γ_e2_ = 0.430, *p* < 0.001; γ_e3_ = 0.452, *p* < 0.001). Affective, behavioral, and continuance commitment played a partial mediating role in the relationship between entrepreneurial intention and entrepreneurial behavior. Thus, H4a, H4b, and H4c were all supported.

The detailed test results of the hypothetical path in the above model are depicted in Table 4.

**TABLE 4:** The result of the path analysis.

Relationship of the hypothetical path	Coefficient of the standardized path	SE	*t*-Value	*p-*Value
Entrepreneurial intention → Entrepreneurial commitments (α_me_)	0.625	0.055	11.328	0.000
Entrepreneurial commitments → Entrepreneurial behavior (β_me_)	0.450	0.076	5.896	0.000
Entrepreneurial intention → Entrepreneurial behavior (γ_me_)	0.256	0.077	3.329	0.001
Entrepreneurial intention → Emotional commitments (α_e1_)	0.530	0.046	11.429	0.000
Emotional commitments → Entrepreneurial behavior (β_e1_)	0.197	0.058	3.378	0.001
Entrepreneurial intention → Entrepreneurial behavior (γ_e1_)	0.427	0.061	7.007	0.000
Entrepreneurial intention → Behavior commitments (α_e2_)	0.333	0.055	6.023	0.000
Behavior commitments → Entrepreneurial behavior (β_e2_)	0.314	0.049	6.464	0.000
Entrepreneurial intention → Entrepreneurial behavior (γ_e2_)	0.430	0.050	8.527	0.000
Entrepreneurial intention → Continual commitments (α_e3_)	0.424	0.061	6.935	0.000
Continual commitments → Entrepreneurial behavior (β_e3_)	0.161	0.064	2.494	0.013
Entrepreneurial intention → Entrepreneurial behavior (γ_e3_)	0.452	0.059	7.659	0.000

### Testing of the Moderated Mediating Effect

The results of the moderated mediating model test illustrated a good fit, with χ*^2^*/*df* = 2.671 (χ*^2^* = 1536.016, *df* = 575), RMSEA = 0.062, CFI = 0.904, and TLI = 0.896. We followed the testing procedure that was proposed by Wen and Ye (2014) to assess the moderated mediation. The moderated mediating model indicated that the entrepreneurial intention (i.e., independent variable) influences the entrepreneurial behavior (i.e., dependent variable) through the entrepreneurial commitment (i.e., mediating variable), and the mediating process is moderated by the family support (i.e., moderating variable). First, we establish a simple moderating model of the relationship between entrepreneurial intention and entrepreneurial behavior to test whether the direct effect is moderated by family support. Next, we establish a moderated mediation model to test whether the mediating effect of entrepreneurial intention on entrepreneurial behavior through entrepreneurial commitment is moderated by family support. Before the test, entrepreneurial intention and family supporting variables were mean-centered to minimize multi-collinearity. We first tested the simple moderating model to investigate whether family support moderates the relationship between entrepreneurial behavior and intention. Entrepreneurial intention significantly predicted entrepreneurial behavior (λ = 0.472, *p* < 0.001), and the interaction effect of entrepreneurial intention and family support was significantly positive (λ*_*if*_* = 0.240, *p* < 0.001).

To further test the role of family support as moderator in the mediating mechanism from entrepreneurial intention to behavior through entrepreneurial commitment, we developed the moderated mediating model with only the indirect effect moderated and conducted path analysis in turn. Results suggest that entrepreneurial intention positively predicted entrepreneurial commitment (α_oe_ = 0.529, *p* < 0.001). The interaction effect of entrepreneurial intention and family support was also significantly positive (λ_ifc_ = 0.136, *p* < 0.05). Entrepreneurial commitment positively predicted entrepreneurial behavior (β_oe_ = 0.366, *p* < 0.001), while the interaction effect between entrepreneurial commitment and family support was not statistically significant (λ_cfb_ = 0.103, *p* = *0.115*). Entrepreneurial intention positively predicted entrepreneurial behavior (γ_oe_ = 0.204, *p* < 0.01), and the interaction effect of entrepreneurial intention and family support was significantly positive (λ_ifb_ = 0.195, *p* < 0.001). The detailed results are depicted in Table 5.

**TABLE 5:** The result of the moderating effect.

Relationship of the hypothetical path	Coefficient of the standardized path	Standard error	*t-*Value	*p*-Value
EI × FS → EB (λ*_*ifb*_*)	0.195	0.048	4.533	0.000
EI × FS → EC (λ*_*ifc*_*)	0.136	0.058	2.335	0.020
EC × FS → EB (λ*_*cfb*_*)	0.103	0.065	1.574	0.115

*EI, entrepreneurial intention; EC, entrepreneurial commitments; FS, family support; EB, entrepreneurial behavior.*

Table 5 shows that family support moderates the mediating effect of entrepreneurial commitment on the link between entrepreneurial intention and behavior. Therefore, H5 and H6 were supported but H7 was not.

**FIGURE 2:**
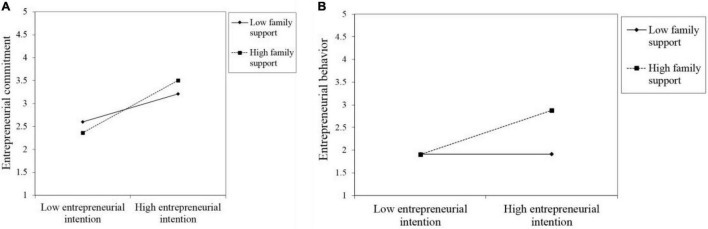
The moderation effect of family support. **(A)** family support moderated the relationship between entrepreneurial intention and entrepreneurial commitment. **(B)** family support moderated the relationship between entrepreneurial intention and entrepreneurial behavior.

To further understand the moderating effect of family support between entrepreneurial intention and behavior, as well as entrepreneurial intention and commitment, we conducted simple slope analyses (Aiken and West, 1991) and plotted the moderating effect of family support in Figure 2. Figure 2A shows the effects of entrepreneurial intention on commitment for two levels of family support: low (regression coefficient was 0.084, *R^2^* = 0.007, *t* = 0.710, *p* = 0.480) and high (regression coefficient was 0.644, *R^2^* = 0.414, *t* = 6.181, *p* < 0.001). It reveals that the relationship between entrepreneurial intention and commitment is stronger at high levels than at low levels of family support, indicating that the positive relationship between entrepreneurial intention and commitment is strengthened by high family support. Figure 2B shows the effects of entrepreneurial intention on behavior for the two levels of family support: low (regression coefficient was 0.236, *R^2^* = 0.056, *t* = 1.986, *p* = 0.051) and high (regression coefficient was 0.578, *R^2^* = 0.335, *t* = 5.354, *p* < 0.001). It reveals that the relationship between entrepreneurial intention and behavior is stronger at high levels than at low levels of family support, indicating that the positive correlation between entrepreneurial intention and behavior is strengthened by high family support. In addition, Figure 2 shows the apparent crossing of two lines in each group. The slope of the solid lines (high family support) is greater than that of the dashed ones (low family support), thus suggesting that entrepreneurial commitment, as well as entrepreneurial behavior, is more strongly associated with entrepreneurial intention when the level of family support is high.

## Discussion

On the one hand, this paper highlighted entrepreneurial commitment and tested the mediating role of its three dimensions including affective, continuance, and behavioral commitment on relationship between entrepreneurial intention and behavior. Some scholars argued that there exists a gap between high entrepreneurial intention and low behavior. Although some studies proposed that entrepreneurial commitment could explain the gap (Wallmeroth et al., 2018), there is currently limited in-depth empirical research on the mechanism. Thus, this study has extended the entrepreneurial cognition theory from intention to behavior. On the other hand, the paper revealed the moderating role of family support on the relationship between entrepreneurial intention, commitment, and behavior, and noted its positive material and psychological effect on entrepreneurs, which implies the potential value of family support for entrepreneurship in the Chinese context.

In the first place, the current study noted the intermediate role of entrepreneurial commitment as a breakthrough in conventional entrepreneurial cognition. It imposes a specific effect underlying the relationship between entrepreneurial intention and behavior through three dimensions of affective, behavioral, and continuance commitment. Compared with entrepreneurial intention (Liu X. et al., 2019), entrepreneurial commitment has closer ties with entrepreneurial behavior (Naz et al., 2020; Sherkat and Chenari, 2020). It was also found that there exists great uncertainty of entrepreneurial intention before it is turned into real behavior merely as an individual’s intention. It mostly remains in thought, so the individual’s decision in entrepreneurship also depends on the transformation and improvement through behavioral strategies. In general, it is through aspects of affection (Dahmardeh and Nastiezaie, 2019; Naz et al., 2020), behavior (Neneh, 2019; Vamvaka et al., 2020), and continuance (Vamvaka et al., 2020) that entrepreneurial commitment imposes a positive influence on the individual’s entrepreneurial intention, which is moderated by family support to encourage individuals to overcome the “gap” between thought and action physically and mentally, and to engage themselves in real entrepreneurship. The commitment with specific aims and plans can influence entrepreneurial behavior development more directly (Wood et al., 2019).

Next, it is worth noting the boundary effect of family support considering the Chinese context in the present study. We argue that the relationship between entrepreneurial intention and behavior is moderated by family support, rather than solely being affected by entrepreneurial commitment. Individuals’ entrepreneurial intention improvement can not only hasten entrepreneurial behavior directly, but also has a positive effect on behavior by improving entrepreneurial commitment. The current study verified that the parents’ cognition of an individual’s entrepreneurship and their material or emotional support are the key to promoting entrepreneurship, as suggested by Zhang and Jia (2016). In addition, family support plays a more important role for individuals with much family support in the development of entrepreneurial commitment and behavior, underpinned by the fact that their entrepreneurial intentions have higher correlations with commitment and behavior. We can infer that in entrepreneurship, which is unpredictable and hard to observe with time lags, support from parents in terms of time, energy, or money, namely, the intervention of family support as the strongest factor in individual decision-making in the Chinese context, can raise potential entrepreneurs’ confidence and sustainability and hence encourage ultimate engagement in entrepreneurship. Although much effort has been devoted to public entrepreneurship, the effect of practice is far from perfect. Given the large variance of individuals’ entrepreneurial abilities and levels, schools or relative social institutions should carry out targeted-entrepreneurial education for individuals suitable for entrepreneurship, further facilitating their behaviors from intentions. On the other hand, for those who are not suitable, their education should focus on entrepreneurial spirit and innovation development, which is essential and indispensable in all fields.

## Conclusion

In this study, we constructed a moderated mediation model to examine whether entrepreneurial commitment from three dimensions (affective, behavioral, and continuance) mediated the relationship between entrepreneurial intention and behavior, and whether this mediating process was moderated by family support. The results of this study verify the decisive role of entrepreneurial commitment underlying the relationship between entrepreneurial intention and behavior. Therefore, individuals cannot only improve entrepreneurial intention directly, but can rely on entrepreneurial commitment as the bridge mechanism to hasten entrepreneurial behavior in order to increase the rate of entrepreneurship in entrepreneurship management. Besides, affective and behavioral commitment plays a more significant role on the link from entrepreneurial intention to behavior as the corresponding mediating effects, which highlights the importance of developing individuals’ affective and behavioral commitment in practice. The study offers evidence indicating that family support strengthens their internal links at both stages from entrepreneurial intention to commitment and from intention to behavior. The result also provides evidence for targeted-entrepreneurial education for individuals with different entrepreneurial intentions, rather than being a unified call for “public entrepreneurship and innovation.” Traditional entrepreneurial education applies to all students and adopts the same methods to encourage their engagement in entrepreneurship, neglecting the variance in their entrepreneurial abilities and levels. Moreover, it is essential to gain a belief of the significance of family support in entrepreneurship in the Chinese context. In order to promote the actual rate of entrepreneurship, we should not solely rely on the efforts of the governments, schools, and society, but also pay attention to the role of family support in the Chinese context, namely, the significant family effect. As a more effective factor, the support from family for entrepreneurs may exert more effects than those from the governments, schools, and the society in entrepreneurship in China.

There are some limitations to be noted in this study: Firstly, there is limited published scales for measuring family support variables currently. The scale adopted in this study was revised from the existing scale on family support. Although the preliminary scale has been tested, it has not been widely used and lacks representativeness. Since family plays an important role in individuals’ major decision making, future research is recommended to further verify and improve the scale of family supports. Secondly, we mainly discussed the mediating effect of the three dimensions of “entrepreneurial commitment.” Future research may consider dividing “entrepreneurial commitment” into three dimensions to analyze its relationship with “entrepreneurial intention” and “entrepreneurial behavior,” which is a more detailed and in-depth study. Finally, this study adopted a cross-sectional approach, which only reflected the relationship between entrepreneurial intention and entrepreneurial behavior at a certain time spot. However, entrepreneurial intention, commitment, and behavior are actually changing over time. Thus, considering the dynamics of entrepreneurship, future research is needed on a longitudinal design in time series to examine the changing nature of the relationship among entrepreneurial intention, commitment, and behavior.

## Data Availability Statement

The original contributions presented in the study are included in the article/supplementary material, further inquiries can be directed to the corresponding authors.

## Ethics Statement

The studies involving human participants were reviewed and approved by South China Normal University. The patients/participants provided their written informed consent to participate in this study.

## Author Contributions

HM identified research ideas, designed and facilitated this research, wrote the draft, and made substantial revisions to this work. ZZ conducted experiments, wrote the draft, assisted in writing the draft, and revised the manuscript. ZM assisted with data collection, analyzed the data, wrote the draft, and provided advice on revisions. WN, HZ, JW, and YH revised the manuscript. All authors contributed to the article and approved the submitted version.

## Conflict of Interest

The authors declare that the research was conducted in the absence of any commercial or financial relationships that could be construed as a potential conflict of interest.

## Publisher’s Note

All claims expressed in this article are solely those of the authors and do not necessarily represent those of their affiliated organizations, or those of the publisher, the editors and the reviewers. Any product that may be evaluated in this article, or claim that may be made by its manufacturer, is not guaranteed or endorsed by the publisher.
